# SARS-CoV-2 infection of human iPSC-derived cardiac cells reflects cytopathic features in hearts of patients with COVID-19

**DOI:** 10.1126/scitranslmed.abf7872

**Published:** 2021-03-15

**Authors:** Juan A. Perez-Bermejo, Serah Kang, Sarah J. Rockwood, Camille R. Simoneau, David A. Joy, Ana C. Silva, Gokul N. Ramadoss, Will R. Flanigan, Parinaz Fozouni, Huihui Li, Pei-Yi Chen, Ken Nakamura, Jeffrey D. Whitman, Paul J. Hanson, Bruce M. McManus, Melanie Ott, Bruce R. Conklin, Todd C. McDevitt

**Affiliations:** 1 Gladstone Institutes, San Francisco, CA 94158, USA.; 2 Biomedical Sciences PhD Program, University of California, San Francisco, CA 94158, USA.; 3 UC Berkeley UCSF Joint Program in Bioengineering, Berkeley, CA 94720, USA.; 4 UCSF Department of Neurology, San Francisco, CA 94143, USA.; 5 UCSF Department of Laboratory Medicine, San Francisco, CA 94143, USA.; 6 University of British Columbia Department of Pathology & Laboratory Medicine, Vancouver, B.C. V6Z 1Y6, Canada.; 7 Innovative Genomics Institute, Berkeley, CA 94704, USA.; 8 UCSF Department of Ophthalmology, San Francisco, CA 94158, USA.; 9 UCSF Department of Medicine, San Francisco, CA 94143, USA.; 10 UCSF Department of Bioengineering and Therapeutic Sciences, San Francisco, CA 94158, USA.

## Abstract

Although coronavirus disease 2019 (COVID-19) causes cardiac dysfunction in up to 25% of patients, its pathogenesis remains unclear. Exposure of human induced pluripotent stem cell (iPSC)-derived heart cells to severe acute respiratory syndrome coronavirus 2 (SARS-CoV-2) revealed productive infection and robust transcriptomic and morphological signatures of damage, particularly in cardiomyocytes. Transcriptomic disruption of structural genes corroborates adverse morphologic features, which included a distinct pattern of myofibrillar fragmentation and nuclear disruption. Human autopsy specimens from patients with COVID-19 reflected similar alterations, particularly sarcomeric fragmentation. These striking cytopathic features in cardiomyocytes provide insights into SARS-CoV-2-induced cardiac damage, offer a platform for discovery of potential therapeutics, and raise concerns about the long-term consequences of COVID-19 in asymptomatic as well as severe cases.

## Teaser

Infection of human iPSC-derived cardiomyocytes by SARS-CoV-2 leads to specific cytopathic features reflected in patient autopsy samples.

## INTRODUCTION

COVID-19, the pandemic disease caused by severe acute respiratory syndrome coronavirus 2 (SARS-CoV-2), was initially characterized as a primarily respiratory syndrome (*1*). However, increasing clinical evidence now implicates multiple organ systems in COVID-19, including the cardiovascular system (heart), digestive system, and urinary system (kidneys) (*2*–*5*). Multiple independent reports have described cases of acute COVID-19-associated myopathy (*6*–*8*) without prior cardiovascular disease (*9*), indicating that SARS-CoV-2 may be directly causing cardiac damage. Meta-analyses identify elevated troponin-I and natriuretic peptides — clinical biomarkers of cardiac damage — as the strongest predictors of mortality in hospitalized patients with COVID-19, eclipsing both prior congestive obstructive pulmonary disease and cardiovascular disease (*8*–*11*). Most hospitalized patients with COVID-19 have abnormal echocardiograms (*12*), and a majority of recovered patients continue to suffer from impaired cardiac function by MRI, indicating that long-term heart sequelae from COVID-19 may not be limited to severe cases of infection (*13*).

Identifying therapeutic strategies to prevent or manage myocardial injury in patients is impeded by a limited understanding of the mechanisms by which SARS-CoV-2 induces cardiac damage. Cardiac damage may be caused by systemic effects of SARS-CoV-2, such as hypoxic stress due to pulmonary damage, microvascular thrombosis, and/or the systemic immune response to viral infection (*14*). However, cardiomyocytes are known to express the primary receptor for viral entry angiotensin-converting enzyme 2 (ACE2) (*15*, *16*) and thus could be infectable by SARS-CoV-2 (*17*, *18*). Viral RNA has been detected in myocardial autopsies of patients infected with SARS-CoV (*19*) and SARS-CoV-2 (*20*), and viral particles have been found within cardiomyocytes and other cardiac cells in patients with COVID-19 (*21*–*23*), suggesting that direct myocardial infection may cause cardiac injury.

Despite the clinical consequences of COVID-19 in the heart, pathological studies of patient autopsy samples have not described specific effects in myocardial specimens, apart from diffuse edema, occasional hypertrophy, and small focal necroses (*24*–*26*). Detailed pathological studies have been hampered by biosafety considerations and the limitations of hematoxylin and eosin (H&E) staining. In addition, sample availability is restricted to post-mortem specimens, which limits most observations to late-stage disease endpoints.

Ex vivo studies using human cell-based models of the heart, such as cardiac tissue derived from human induced pluripotent stem cells (iPSCs), afford the most direct route for prospective and clinically relevant studies on the effects of cardiac viral infection. Stem-cell derived models have already demonstrated the susceptibility of hepatocytes (*27*), intestinal epithelium (*28*, *29*), and lung organoids (*30*) to SARS-CoV-2 infection. While two recent reports confirmed that human iPSC-cardiomyocytes are susceptible to SARS-CoV-2 infection (*31*, *32*), specific cardiac cytopathic features have yet to be identified. In addition, the viral tropism for other cardiac cell types, which may be involved in microthromboses (*33*) or weakening of the ventricular wall, has not been explored, nor has there been direct correlation of in vitro results to clinical pathology specimens. Here, we examined the relative susceptibility to SARS-CoV-2 infection of three cardiac cell types derived from iPSCs: cardiomyocytes (CMs), cardiac fibroblasts (CFs), and endothelial cells (ECs). We observed hallmarks of infection and cardiac cytopathy that led us to identify pathologic features in human COVID-19-infected cardiac tissue specimens. The high infectability of iPSC-CMs by SARS-CoV-2 and the observed phenotypic biomarkers of infection could potentially enable a discovery platform for the development of therapeutic and cardioprotective approaches for COVID-19.

## RESULTS

### SARS-CoV-2 productively infects human cardiomyocytes, but not endothelial cells or fibroblasts

The relative susceptibility of different cardiac cell types to SARS-CoV-2 infection has not been characterized, leading to ambiguity regarding the sources of cardiac damage and relevant therapeutic targets. Analysis of single-cell RNA-sequencing and immunofluorescence staining of iPSC-ECs, -CFs, and -CMs revealed that transcripts for ACE2, the receptor for SARS-CoV-2 entry, were only detectable in CMs **(fig. S1, A to D)**. Although expression of the cell surface protease TMPRSS2, which is commonly involved in viral cell entry, was not detected in any cell type, expression of cathepsin-L (CTSL) and cathepsin-B (CTSB) was detected in all cells. These observations support the potential infectivity of CMs by SARS-CoV-2, and predict poor infectivity in ECs and CFs.

**Fig. 1:**
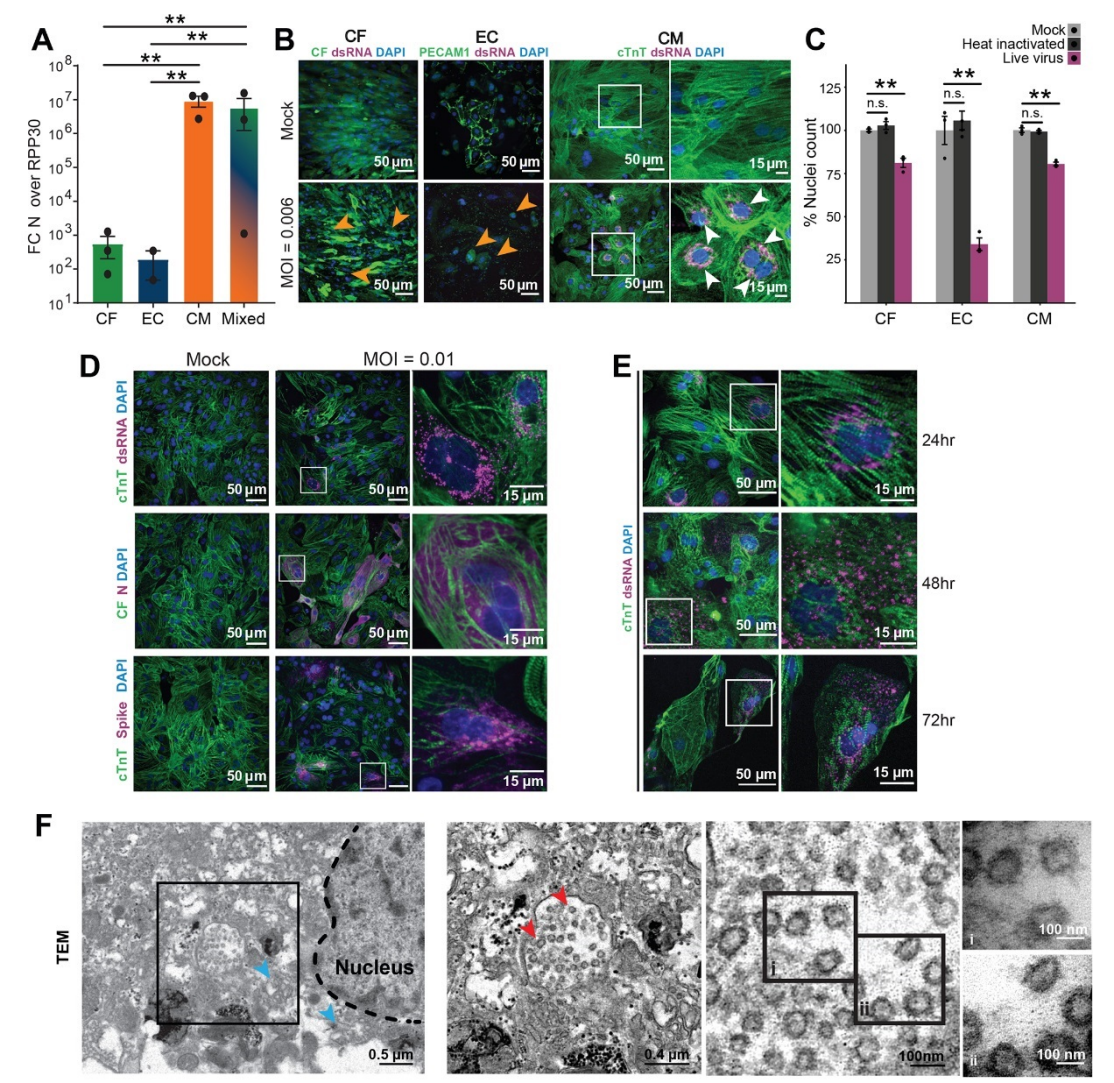
**Effects of SARS-CoV-2 exposure on different iPSC-derived cardiac cell types**. In all experiments, cells were exposed to SARS-CoV-2 virus for 48 hours at an MOI of 0.006 before lysis or fixation, unless otherwise specified. White and black boxes indicate magnified regions. (**A**) RT-qPCR quantification of viral RNA [Fold change (FC) of SARS-CoV-2 nucleocapsid (N) gene, N5, over housekeeping gene transcript, RPP30] in cell cultures exposed to SARS-CoV-2. CF: iPSC-derived cardiac fibroblasts; EC: iPSC-derived endothelial cells; CM: iPSC-derived cardiomyocytes; Mixed: 60:30:10 CM:EC:CF. Bars: mean. Error bars: SEM. **P < 0.01, one-way ANOVA with Tukey’s multiple comparisons. technical replicates: 3; *N* = 3. (**B**) Representative images of immunostaining of cardiac cells exposed to SARS-CoV-2. PECAM-1 (CD31) was used as an EC marker, and cTnT as a CM marker. CFs expressed GFP constitutively. Viral signal was detected by staining for SARS-CoV-2 spike protein or viral double stranded RNA (dsRNA), as noted. White boxes represent regions magnified in rightmost panels. Orange arrowheads indicate seemingly apoptotic bodies and white arrowheads denote clusters of dsRNA signal. Images are selected from a total of 30 images across 3 replicates. (**C**) Toxicity of SARS-CoV-2 to cardiac cell types, quantified by nuclear retention. Y-axis depicts the % of nuclei counted (relative to mock). Nuclei were counted automatically at 10x magnification (10 images/condition). Light gray: Vehicle treatment (mock), Dark gray: Heat inactivated SARS-CoV-2 (MOI = 0.1), Magenta: SARS-CoV-2 (MOI = 0.006). Bars: mean. Error bars: SEM. **P < 0.01. n.s.: nonsignificant (*P* > 0.05). *n* = >500 cells per group. *N* = 3. (**D**) Representative images of immunostaining for different SARS-CoV-2 viral antigens (dsRNA, N protein or spike protein) in infected iPSC-CMs. Images are selected from a total of 30 images across 3 replicates. (**E**) Representative images of immunostaining of infected CMs at 24h, 48h or 72h after addition of SARS-CoV-2 virus. Images are selected from a total of 30 images across 3 replicates. (**F**) Transmission electron microscopy of SARS-CoV-2 viral particles in an infected CM. Left: Montage view with nucleus (dashed line), in addition to remnant ER-Golgi (light blue arrowheads), with viral particles enclosed in a membrane compartment. Middle-left: Magnified view of boxed region from leftmost panel showing SARS-CoV-2 virions (red arrowheads) and surrounding membrane. Middle-right: Magnified view of SARS-CoV-2 virions, showing the 500-750 nm diameter membrane and the 60-100 nm diameter viral particles within. Right: High magnification images of indicated regions of interest within adjacent panel (denoted **i** and **ii**). Images are selected from a total of 55 images across 3 different cells.

To validate the gene expression predictions, we exposed human iPSC-derived CMs, CFs, ECs, or a mixture of all three cell types to mimic native myocardial composition, to SARS-CoV-2 at a low multiplicity of infection (MOI) (0.006). After 48 hours, CFs and ECs showed little to no viral RNA relative to a housekeeping control, whereas CMs expressed >10^4^ greater amounts of viral RNA than CFs or ECs **(****Fig. 1A****)**. There was no significant (*P* > 0.05) difference in viral detection between CMs and mixed cultures **(****Fig. 1A****)**, and undifferentiated iPSCs were uninfectable **(fig. S1D)**. Differences in viral RNA detection largely corresponded with cell-type specific ACE2 expression **(fig. S1, A and B)**. To confirm that detected viral titers resulted in the production of infective virions, we performed plaque assays with the supernatants of virus-exposed cells. CFs, ECs, and iPSCs did not support productive infection, whereas CMs produced replication-competent virions **(fig. S1E,** note that, for iPSC-CM, number of plaque-forming units (PFU) is lower than the number of infected cells, possibly due to the low MOI used (0.006) used or wash-off of virions released within the first 24 hours).

Immunostaining for viral double-stranded RNA (dsRNA), a viral intermediate produced during active replication of single-stranded RNA viruses (*34*), further confirmed that CMs, but not CFs or ECs, supported viral RNA replication **(****Fig. 1B****)**. However, all three cell types exhibited marked cytopathic effects after 48 hours of viral exposure, characterized by fragmented cell bodies, dissociation from neighboring cells, and significant cell death **(****Fig. 1C**, *P* < 0.05**)**. Visual cytopathic effects were most prevalent in CFs, while the greatest nuclear loss was observed in ECs, indicating that toxicity from viral exposure can occur without detectable viral replication. However, inoculation with heat-inactivated SARS-CoV-2 did not cause cell death or dissociation in any of the cell types assayed **(****Fig. 1C****)**. Infectivity of CMs was further validated by staining for viral nucleocapsid (N), which showed intense uniform signal over the cell body, and spike (S) protein, which primarily exhibited localization to sub-micrometer-sized aggregates that resembled intracellular vesicles **(****Fig. 1D****)**.

Replication of positive-strand single stranded RNA viruses, including SARS-CoV-2, involves budding of double-membrane vesicles from the endoplasmic reticulum (ER), with viral particle assembly occurring in cisternae of the ER-Golgi intermediate compartment (ERGIC) (*35*). In CMs infected with SARS-CoV-2, dsRNA and spike protein initially (24h post-infection) accumulated around the nucleus in small perinuclear puncta, the typical location of the ERGIC, indicating potential replication centers **(****Fig. 1E****)**. At 48h post infection, many cells exhibited dsRNA signal dispersed throughout their cytoplasm, which may correlate with advanced stages of infection. By 72h post infection, SARS-CoV-2 was spread throughout the culture and a large portion of the CMs had died, with the remaining cells displaying disperse viral stain localization, dissociation from neighboring cells, and heavily reduced sarcomeric staining **(****Fig. 1E****)**. To study the effects of increased viral dose, we infected CMs at two different MOIs and observed a correlation between inoculated virus, viral transcripts, and virus-positive cells **(fig. S1, F to G)**. Infection at a higher MOI (0.1) resulted in a uniform appearance of virus-positive cells by staining, whereas a lower MOI (0.01) resulted in localized foci of infection. The regional clusters of viral infection resulted in high variability in the low MOI condition **(fig. S1, G to H)**, suggesting amplification of a small number of initial infection events (*36*). This was further analyzed by quantification of the viral infection rate, which revealed marked variability even at a consistent MOI (compare **fig. S1, F and G**).

Remnants of the ER-Golgi membranes and large vesicles near the nucleus in infected CMs were readily identified by transmission electron microscopy (TEM) **(****Fig. 1F**). These vesicles, about 500-750 nanometers (nm) in diameter, contained multiple particles 60-100 nm in diameter that were identified as SARS-CoV-2 virions **(****Fig. 1F****)**. Recently, similar vesicles loaded with mature virions have been identified as vacuoles or deacidified lysosomal compartments used by SARS-CoV-2 for viral egress (*37*, *38*). These results indicate that SARS-CoV-2 is able to infect, replicate in, and rapidly propagate among human CMs.

### SARS-CoV-2 infection of cardiomyocytes is dependent on endolysosomal entry

**Fig. 2:**
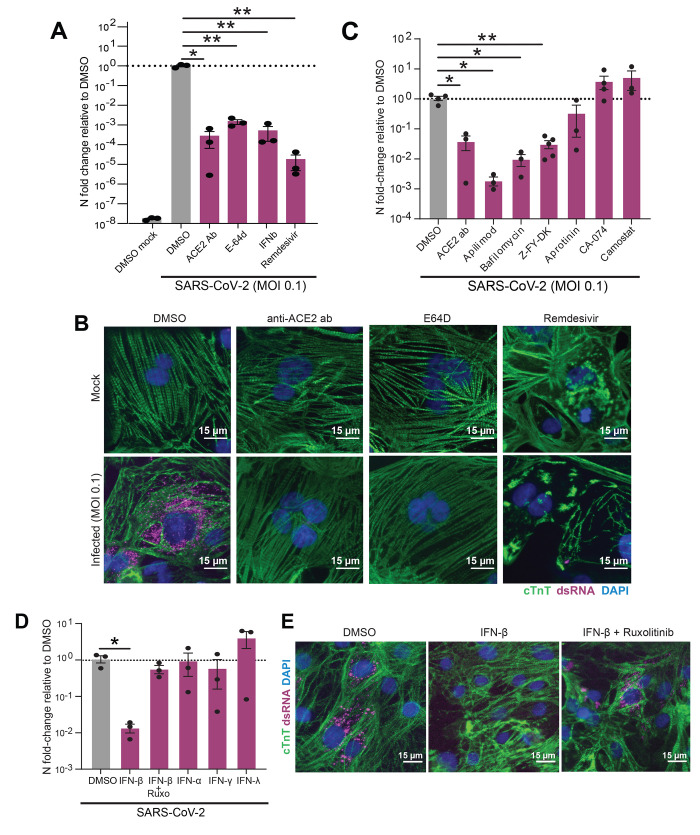
Pharmacological modulation of SARS-CoV-2 infection, viral entry and innate immune response in CMs. (**A**) RT-qPCR quantification of viral RNA (N5) in CM samples exposed to SARS-CoV-2 for 48h (MOI = 0.1) after 2h pretreatment with the indicated reagents to block viral infection. Bars: mean. Error bars: SEM. *P < 0.05,**P < 0.01, one-way ANOVA with Tukey’s multiple comparisons. Technical replicates: 3; *N* = 3. (**B**) Representative immunofluorescence images from SARS-CoV-2-infected (MOI = 0.1) CMs pretreated with either vehicle (DMSO), ACE2 blocking antibody (anti-ACE2 ab), cathepsin-B and -L inhibitor E-64d (E64D) or remdesivir for 2h before infection. Double-stranded RNA (dsRNA) staining (magenta) denotes presence of replicating virus. Images are selected from a total of 30 images across 3 replicates. (**C-D**) RT-qPCR quantification of viral RNA (N5) in CM samples exposed to SARS-CoV-2 for 48h (MOI = 0.006) after 2h pretreatment with the indicated reagents to block viral entry (**C**) or prime the cells’ innate immune response (**D**). Dots represent separate replicates. Bars: mean. Error bars: SEM. *P < 0.05, **P < 0.01, one-way ANOVA with Tukey’s multiple comparisons. Technical replicates: 3; *N ≥* 3 for all conditions. Z-FY-DK: Z-Phe-Tyr(tBu)-diazomethylketone, specific Cathepsin-L inhibitor; CA-074: specific Cathepsin-B inhibitor; Ruxo: Ruxolitinib, JAK1/2 inhibitor. (**E**) Representative immunofluorescence images from cardiomyocytes pre-treated with vehicle (DMSO), IFN-β, or IFN-β with JAK inhibitor ruxolitinib. Images are selected from a total of 30 images across 3 replicates.

We next sought to examine clinically relevant strategies to prevent SARS-CoV-2 infection of CMs. Pre-treatment of cells with an ACE2 blocking antibody, with the cathepsin-B/-L inhibitor E-64d, or with the antiviral drug remdesivir significantly reduced viral detection in infected CMs **(****Fig. 2A****,** *P* < 0.01**)**. Although CMs express the furin protease, small molecule inhibition of FURIN did not reduce infection **(fig. S1I)**. Inhibition of viral infection generally prevented cytopathic effects, although remdesivir showed marked toxicity to CMs at the concentration used (10 μM), in agreement with a previous report (*39*) **(****Fig. 2B****)**. To further dissect the mechanism of SARS-CoV-2 infection of CMs, we observed that specific CTSL inhibition via Z-Phe-Tyr(tBu)-diazomethylketone (Z-FY-DK) decreased viral detection in infected cells to about 10% of vehicle-treated controls, whereas inhibition of CTSB with CA-074 did not attenuate viral RNA detection **(****Fig. 2C****)**. Furthermore, the PIKfyve inhibitor apilimod and autolysosome acidification blocker bafilomycin each successfully reduced viral infection to ~0.1% or 1% of vehicle-treated controls, respectively. In contrast, the TMPRSS2 inhibitors aprotinin and camostat mesilate did not significantly inhibit viral infection (*P* > 0.1). Collectively these results indicate that SARS-CoV-2 binds to iPSC-CMs via the ACE2 receptor, and uses a CTSL (but not CTSB)-dependent endolysosomal route of entry and/or egress, independent of TMPRSS2/serine protease-mediated activation at the cellular membrane (*37*).

We next examined whether priming the innate immune response could reduce SARS-CoV-2 infection of CMs. CMs were treated with interferon alpha (IFN-α), beta (IFN-β), gamma (IFN-γ), or lambda (IFN-λ) prior to infection. Only pre-exposure to IFN-β decreased infection, and this effect was reversed by co-administration of the JAK/Stat inhibitor ruxolitinib **(****Fig. 2, D to E****)**, confirming that CMs can mount an antiviral response with appropriate stimulation. Our observation that IFN-β but not IFN-α pre-treatment reduced infection is in concordance with a previous report of differential antiviral activity of these two type-I interferons in a mouse model of myocarditis (*40*).

### SARS-CoV-2 exposure induces broad transcriptional changes

**Fig. 3:**
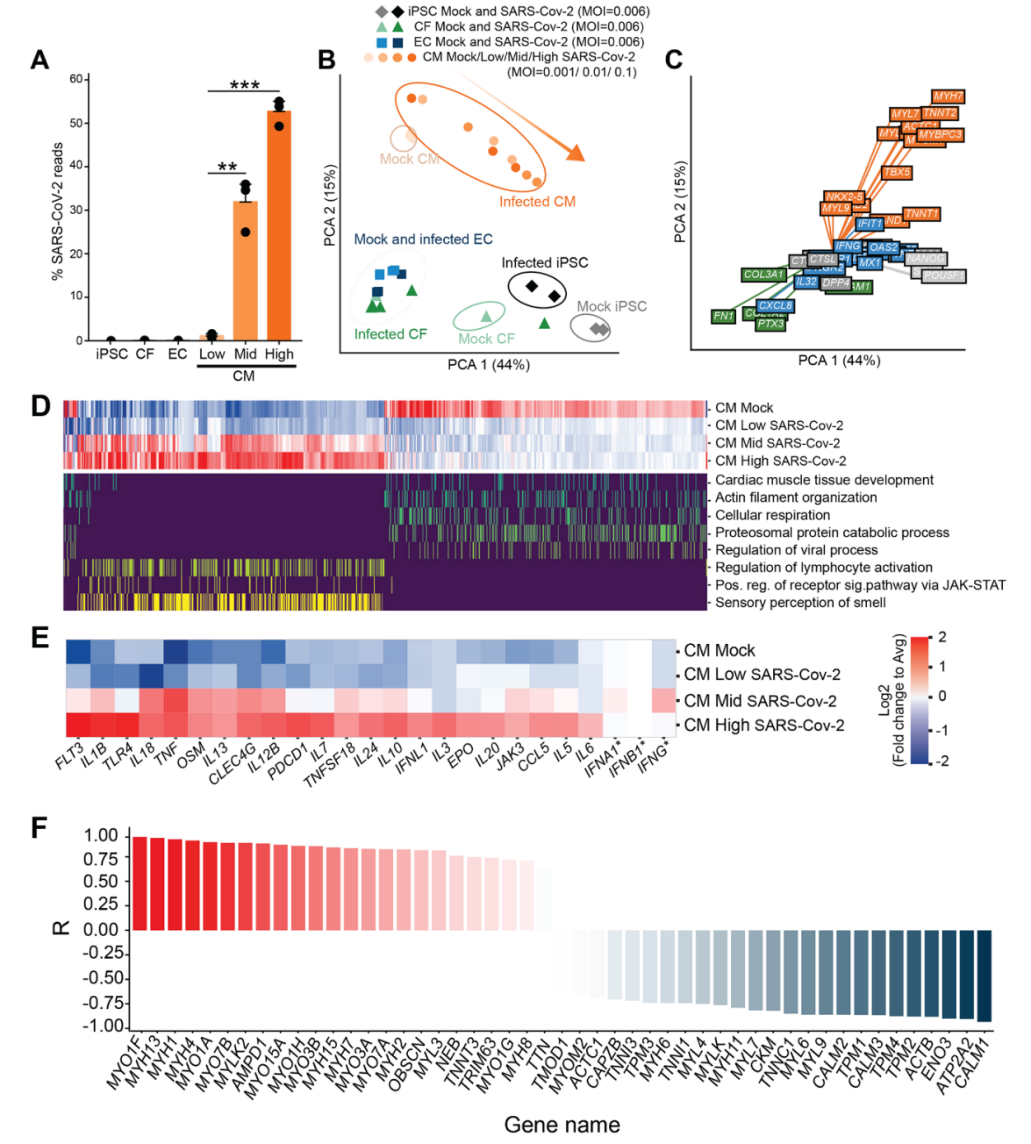
Transcriptional effects of SARS-CoV-2 exposure to cardiac cells. (**A**) Percentage of total reads that map to the SARS-CoV-2 viral genome in various cell types. iPSCs, ECs or CFs were exposed at an MOI of 0.006, and CMs were exposed at three different MOIs: 0.001 (Low), 0.01 (Mid) and 0.1 (High). Bars: average %reads. Error bars: SEM. **P < 0.01; ***P < 0.001. one-way ANOVA with Tukey’s multiple comparisons. Technical replicates: 3; *N* = 3. (**B).** Principal component analysis of transcriptomic samples. Dot shapes and colors represent the different cell types, whether they were exposed to SARS-CoV-2 virus and, in the case of CMs, the different MOIs used. Orange arrow indicates increasing degrees of transcriptional dysregulation (relative to Mock). *N* = 3. (**C).** Loading plot for a selected subset of genes, with color indicating cardiomyocyte state (orange), fibroblast/endothelial cell state (green), iPSC state (light gray), SARS-CoV-2 infection-related factors (dark gray), immune response (blue). *N* = 3. (**D).** Heat map depicting transcriptional expression profiles for genes mapping to GO terms of interest. Top: expression profile in the Mock condition and least (Low), middle (Mid), and most (High) transcriptionally disrupted CM samples. Bottom: mapping of genes to specific GO terms of interest (only yes/no scale). All genes in the plot had |log2 fold change| > 1 between high infection and mock, adjusted false discovery rate (FDR) < 0.05 using edgeR’s differential expression test (see Methods section); *N* = 3. All GO terms selected contained at least 25 enriched genes, adjusted FDR < 0.01, using an enrichment test based on hypergeometric distribution and controlled FDR (see Methods). (**E).** Zoomed in heat map depicting differential expression profiles for a select number of genes involved in innate and inflammatory response to viral infection. All genes are regulated in a statistically significant manner (adjusted *FDR* < 0.05) except for *IFNA1, IFNB1*, and *IFNG* (indicated by an asterisk). Color represents log-2 fold change from average value across conditions. *N* = 3. Color scale represents log2 fold change relative to average expression of all conditions. **(F).** Expression ratio (R) of genes involved in sarcomeric structure and myosin contractility of the high-infection CM groups relative to the mock-infection CM group. Expression ratio is the Pearson correlation of gene expression with increasing level of infection. *N* = 3. Fisher-transformed *P* < 0.01.

To evaluate the transcriptional impact of SARS-CoV-2 on cardiac cells, we performed RNA-sequencing of CFs, ECs, and iPSCs exposed to an MOI of 0.006 and CMs exposed to a range of MOIs (0.001, 0.01, and 0.1). Sequencing recovered SARS-CoV-2 reads in an MOI- and cell type-dependent fashion **(****Fig. 3A****)**, with SARS-CoV-2 reaching >50% of the recovered reads in CMs at the highest MOI. Principal component analysis (PCA) of the biological conditions revealed clustering based primarily on cell type, with CFs and ECs clustering together while CMs and iPSCs separated into distinct clusters **(****Fig. 3B****)**. Loading plots of the principal components supported this interpretation: genes determining the spectrum of variation between CMs and CFs/ECs were associated with CMs (*MYH7*, *MYH6*, *TNNT2*) at one pole **(****Fig. 3C****)** and anti-correlated with CFs/ECs-specific genes at the other (*FN1*, *COL1A2*, *TFPI2*, *MME*) **(fig. S2A)**. However, the distance between mock-treated CMs and the furthest infected CMs was greater than the distance between CMs and CFs or ECs, indicating that viral infection altered cellular expression profiles at least as strongly, if not more so, than cell type. Along this PCA axis, the extent of transcriptional disruption correlated poorly with MOI across all CM samples, potentially due to natural stochasticity in the kinetics of infection. This variability of parallel infections for low MOIs is in agreement with our observations of high variability in number of cells infected by imaging for parallel replicate infections. However, regrouping conditions by the relative degree of transcriptional disruption allowed for clearer deduction of transcriptional trends resulting from viral exposure **(fig. S2, A to D)**.

The differentially regulated genes involved in inflammation and innate immunity reflected the observed preferential infectivity of CMs. Exposed CFs and ECs had a depressed cytokine response compared to CMs at all three MOIs examined **(fig. S2E),** while infected CMs were enriched in genes involved in inflammatory cytokine production and T-cell activation, such as *IL6, IL1B, TNF, CCL5/RANTES, EPO, OSM* and others **(****Fig. 3D and E****, fig. S2E)**. Interferon gene expression was not significantly changed in any condition, suggesting active repression by SARS-CoV-2 as previously reported (*41*) **(****Fig. 3E****,** adjusted *P* > 0.05**)**.

Infected cardiomyocytes down-regulated pathways corresponding to cardiac muscle tissue organization and cellular respiration **(****Fig. 3, D and F****; fig. S3, A to C)**. Anomalous up-regulation of pathways associated with olfactory receptors and dysregulation of proteasome catabolism were also observed with increasing transcriptional disruption **(****Fig. 3D****, fig. S2E)**. CMs at each MOI showed clear dysregulation of contractile machinery, proteasomal subunits and ubiquitination (**fig. S3, A to D**). Genes involved in the Linker of Nucleoskeleton and Cytoskeleton (LINC) complex were disrupted, particularly calmin and members of the nesprin family, both critical for anchoring the nucleus to the actin cytoskeleton **(fig. S3E; fig. S4, A and B)**. Furthermore, sarcomeric structural proteins, myosin light chains, and proteasome kinases and chaperones were strongly down-regulated, whereas most myosin heavy chains were significantly up-regulated **(****Fig. 3F****; fig. S3, A and B; fig. S4C;** adjusted *P* < 0.05**)**, suggesting a divergent effect of SARS-CoV-2 infection on the contractile and structural integrity of CMs.

### SARS-CoV-2 infection disrupts multiple intracellular features of cardiomyocytes

**Fig. 4:**
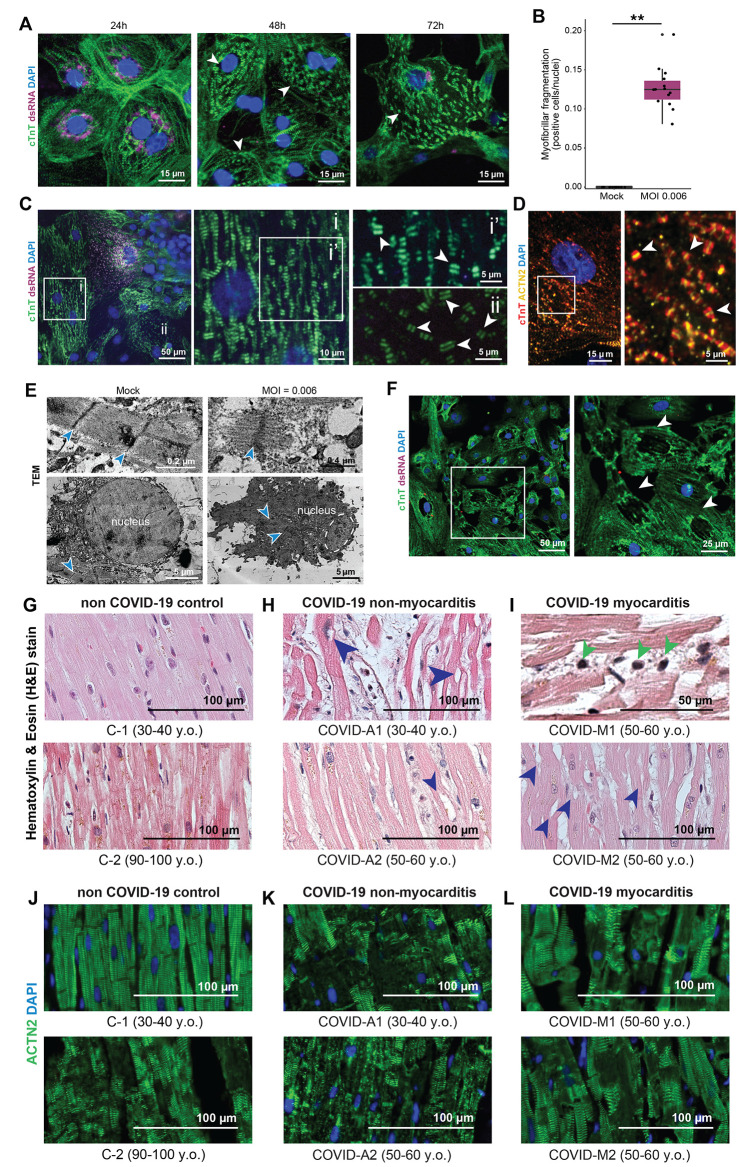
Analysis of cytopathic features in iPSC-derived CMs infected by SARS-CoV-2 and autopsy myocardial tissue from healthy individuals and patients with COVID-19. (**A**) Representative immunofluorescence images of myofibrillar fragmentation in CMs at different timepoints after exposure to SARS-CoV-2. White arrowheads indicate fragments consisting of two bands of cTnT+ staining. Images are selected from a total of 30 images across 3 replicates. (**B**) Quantification of number of cells presenting myofibrillar fragmentation at 48h post-exposure (defined as cells presenting at least one event of a cTnT doublet unaligned and dissociated from other myofibrils, divided by total nuclei count). Each dot represents a separate infected sample, each one being the sum of 9 randomly acquired fields of view. **P < 0.01. Two-tailed *t* test after checking for normality. (**C**) Representative immunostaining showing a cell with viral dsRNA, adjacent to cells with different degrees of myofibrillar fragmentation. White squares indicate areas magnified in right panels, with labels corresponding to insets. White arrowheads point to examples of cTnT doublets (myofibrillar fragments). Images are selected from a total of 55 images across 3 different cells. (**D**) cTnT and ACTN2 double-staining of CMs displaying myofibrillar fragmentation. White arrowheads indicate cTnT-ACTN2-cTnT myofibrillar fragments. (**E**) TEM images of sarcomeres in mock-treated and SARS-CoV-2-infected (MOI = 0.006) CM cultures. Blue arrowheads denote the sarcomeric z-disk; Yellow arrowheads indicate M-line location; dashed line delimits nucleus. Sarcomeres of mock-treated cells display clear I and A-bands, but fragmented sarcomeres only possess thin filaments. Below: Representative TEM image of a healthy nucleus, and the nucleus of a cell infected with SARS-CoV-2. (**F**) Immunofluorescence staining of SARS-CoV-2-exposed CMs displaying loss of nuclear DNA staining (48h post exposure). White arrowheads indicate locations of sarcomeric retraction and absence of nuclear material. Images are selected from a total of 30 images across 3 replicates. (**G-I**) Representative images of Hematoxylin and Eosin (H&E) staining of myocardial tissues from patients without COVID-19 (**G**), and patients with COVID-19 without cardiac involvement (**H**) or with diagnosed myocarditis (**I**). Red arrowheads indicate cardiomyocytes lacking chromatin staining. Dark blue arrowheads indicate the presence of immune cells. (**J-L**) Representative immunofluorescence staining of myocardial tissue from patients without COVID-19 (**J**), compared to patients with COVID-19 without cardiac involvement (**K**) and with diagnosed myocarditis (**L**). Cardiomyocytes show signs of damage in the form of diffuse and disorganized actinin (ACTN2) staining. For all patient biopsies, images are selected from 2-3 heart regions (right and left ventricles and interventricular septum) per sample, 5-15 images per section/region, for a total of 7 patients (two control, two with COVID and diagnosed myocarditis, and three with COVID and no diagnosed myocarditis).

Motivated by the expression changes in structural and contractile genes in our transcriptomic data, we performed high-content imaging of CMs following SARS-CoV-2 infection. Several abnormal structural features were observed in infected CMs that were not seen in mock or heat-inactivated virus-treated CMs, including widespread myofibrillar disruption throughout the cytoplasm: a unique pattern of specific, periodic cleavage of myofibrils into individual sarcomeric units of identical size without any linear alignment **(****Fig. 4A to C****; fig. S5, A to D)**. Myofibrillar fragmentation was present in up to 20% of the cells exposed to SARS-CoV-2, and, similar to the previously noted inconsistencies of infection, displayed with variable prevalence across experiments (6-20% incidence rate captured moment; multiple fragments per cell) **(****Fig. 4D****, fig. S5E)**. Fragmentation was observed as early as 24 hours after infection, and significantly increased after 48 hours of viral exposure, suggesting progression over the course of infection **(fig. S5E,** *P* < 0.05**)**. Curiously, this pattern of myofibrillar fragmentation was present in cells independent of actively replicating virus (as per dsRNA staining; Chi-square test for independence *P* = 0.81) **(****Fig. 4C****).**

Co-staining of CMs with the thin filament marker cardiac troponin T (cTnT) and the Z-disk marker α-actinin 2 revealed that myofibrillar fragments induced by SARS-CoV-2 consisted of two bands of cTnT flanking a single α-actinin 2 band **(****Fig. 4D****, fig. S5F)**. Such cells exhibited significant cytotoxic stress, as evidenced by collapse of their mitochondrial networks **(fig. S5G)**. To examine sarcomeric fragmentation in greater detail, we employed TEM imaging of SARS-CoV-2-infected and mock-treated CMs. While intact sarcomeres were clearly identified with a classic dark Z-disk adjacent to a light I-band followed by a dark A-band, single fragmented myofibrils displayed an extended I-band and complete absence of the A-band **(****Fig. 4E****)**, indicating a liberation of thick filaments from sarcomere subunits.

To explore specificity of this sarcomeric fragmentation phenotype, CMs were exposed to other coronaviruses, NL63 and OC43. Both resulted in successful infection, but did not induce a comparable myofibrillar phenotype, suggesting this cytopathic effect is specifically induced by SARS-CoV-2 infection **(fig. S6A)**. Since transcriptomic profiling indicated that viral infection perturbed the proteasome system **(****Fig. 3F****, fig. S3F)**, we also examined whether proteasome inhibition could recapitulate similar structural abnormalities. Although high doses of the proteasome inhibitor bortezomib induced myofibril fragmentations in CMs, the effect was much less prevalent and less severe (<1% prevalence, only a few fragments per cell) than the effect of SARS-CoV-2 infection, and was generally accompanied by diffuse cTnT staining **(fig. S6B)**. Furthermore, the well-known cardiotoxic drug doxorubicin did not induce myofibril fragmentation **(fig. S6B)**, suggesting that proteasomal inhibition may specifically recapitulate part of the viral effects that lead to myofibrillar fragmentation. We also observed a second structural phenotype in which some CMs from infected cultures often lacked nuclear DNA staining. This phenomenon was characterized by withdrawn sarcomeres and abnormally shaped or absent nuclear DNA signal **(****Fig. 4F****)**. Both phenotypes of sarcomeric fragmentation and abnormal nuclear structures are consistent with our observed transcriptomic disruptions, corresponding with disruption of thick filament-specific genes and dysregulation of the LINC complex, respectively (**fig. S4**). The transcriptional changes could reflect a compensatory response to depletion of these structural proteins due to the action of the virus.

### In vitro findings mirror disruptions in myocardium of patients with COVID-19

We next asked whether the SARS-CoV-2 induced phenotypes observed in CMs in vitro reflected similar patterns of cardiac cell damage in vivo. We obtained autopsy specimens from five patients diagnosed with COVID-19 prior to death: two who had been diagnosed with myocarditis (COVID-M1 and COVID-M2), and three that had no reported cardiac involvement (COVID-A1, COVID-A2 and COVID-A3). We also obtained samples from two patients without COVID-19, which served as age-matched controls (C-1 and C-2). Compared to non-COVID-19 controls (Age-matched: **Fig. 4G****;** Neonatal: **fig. S7A**), patients with COVID-19 displayed disrupted myocytes with visible loss of DNA staining (**Fig. 4, H and I****; fig. S7, B and, C**) regardless of myocarditis, across five independent sections from different tissue regions. A minor difference in nuclear counts (~10%) between regions of intact and disrupted tissue was observed across samples from patients with COVID-19 (**fig. S7D**). Diagnosed myocarditis patients’ hearts also displayed infiltration of mononuclear immune cells (**Fig. 4I**), in addition to occasional troponin-positive mononuclear cells in the vasculature (**fig. S7B**).

Similar to previous studies of COVID-19 autopsy samples (*20*, *42*–*44*), hematoxylin and eosin staining did not reveal signs of myofibrillar damage, so we further probed the autopsy samples by immunostaining for sarcomeric proteins. All samples from patients with COVID-19 presented severe myofibrillar anomalies in the form of diffuse or absent sarcomeric protein staining (cTnT and α-actinin-2). In particular, compared to control, COVID-A1, A2 and A3 **(****Fig. 4, J and K****, fig. S7C)** displayed a clear pattern of disruptions to the sarcomeric banding, while COVID-M1 and M2 displayed diffuse staining in myocytes **(****Fig. 4L****)**. These aberrant features moderately resembled those observed in the oldest COVID-19 free control specimen (>90 yrs old) **(****Fig. 4J****).** We also attempted to identify viral presence in the tissues using antibodies against SARS-CoV-2 spike protein, dsRNA, and two different epitopes of the viral nucleocapsid protein, but were unable to detect viral signal in any autopsy patient tissues. Overall, these patterns suggest that myocarditis in the heart leads to tissue damage, but is not a requirement for myofibrillar disruption of cardiac tissue, and that the aberrant features observed in the hearts of patients with COVID-19 are pathologic in nature and bear similarities to observations in the oldest specimen. Mild infiltration of mononuclear immune cells is occasionally noted in non-myocarditis cases, and may contribute to elements of the in vivo response. The patterns of myocardial damage identified in heart specimens from deceased individuals with COVID-19 heart specimens closely resemble similar cytopathic features first identified by in vitro exposure of iPSC-CMs to SARS-CoV-2.

## DISCUSSION

Here, we conducted a comprehensive analysis of the cytopathic effects of SARS-CoV-2 in human iPSC-derived cardiac cells to model viral infection of the heart. Cytopathic effects were particularly striking in CMs, which manifested a distinctive pattern of myofibrillar fragmentation into individual sarcomeric units and a loss of nuclear DNA staining from intact cell bodies. Surprisingly, these cytopathic effects occurred independent of the presence of actively replicating SARS-CoV-2 virus, suggesting a broader spectrum of adverse consequences than initially assumed. Guided by these observations, we observed similar sarcomeric structural disruptions in the myocardium of deceased patients with COVID-19. Together, these results provide insights into the pathogenesis of SARS-CoV-2-induced heart damage, indicate new avenues for the development of cardioprotective interventions against COVID-19, and raise concerns about the prevalence and severity of cardiac involvement due to COVID-19 disease.

Determining the mechanisms responsible for diminished cardiac function is critically important to developing cardioprotective therapies for COVID-19. We observed that SARS-CoV-2 infection creates precise and ordered disruptions to the myofibrillar structure and dissolution of the cardiac contractile machinery, which would inevitably lead to functional collapse. The prevalence of these effects varied, but was observed to be as high as 20% at a single time point in our in vitro experiments. The striking consistency and periodicity of fragmentation suggests separation of the sarcomeric thick and thin filaments, as shown by immunofluorescence and TEM, perhaps due to cleavage by a specific protease. Additionally, our transcriptomic analyses showed a compensatory overexpression of myosin heavy chain genes in response to this degradation. Myosin heavy chain family members contain the LKGG↓K sequence, which matches one of the sites used by the viral papain-like protease (PLPro) to cleave the viral polyprotein (*45*, *46*). Such cleavage of myosin could result in the observed filament-specific separation (*47*), although this hypothesis would not immediately explain why myofibrillar disruption was present in cells exposed to SARS-CoV-2 independently of dsRNA signal. Indeed, cytopathic effects independent of dsRNA signal suggests a range of possible explanations, including abortive infection, autonomous cellular chemokine secretion, or multiple distinct stages of infection.

Depression of the ubiquitin-proteasome system was also observed upon SARS-CoV-2 infection. Myofibrillar fragmentation could be partially recapitulated by proteasomal inhibition, but not by other cardiotoxic drugs or by other coronavirus infection of CMs. Proteasomal homeostasis is critical for maintenance of cardiac function (*48*), and dysregulation of protein quality control and translation by SARS-CoV-2 may induce degradation of sarcomeres that results in the observed myofibrillar fragmentation phenotype. The non-concomitance of myofibrillar fragmentation and actively replicating virus could be suggestive of unsuccessful infection (*36*) or a bystander effect. While further interrogation is needed to determine the precise mechanisms mediating myofibrillar fragmentation, the CMs pathologic phenotype still informs expectations for histological specimens and therapeutic discovery.

To date, most myocardial histology from autopsy specimens of patients with COVID-19 have revealed only general signs of myopathy, such as edema, occasional mononuclear infiltrate, and mild hypertrophy (*20*). Although 30-50% of individuals with COVID-19 manifest clinical signs of cardiac dysfunction (*13*, *49*), histological hallmarks of COVID-19 in the heart have remained elusive. Our preliminary examinations of hematoxylin and eosin staining of COVID-19 myocardial samples revealed only minor disruption and generally intact myofibrillar anatomy. However, guided by our in vitro analysis, we performed immunostaining for sarcomeric proteins to identify clear features of myocardial damage in COVID-19 autopsy samples. The pattern of sarcomere staining loss was consistently found in all the COVID-19 samples, and only moderately resembled a single control sample from a much older patient. This observation reveals the potential underlying etiology of SARS-CoV-2’s impact on cardiac function and demonstrates that human iPSC-derived models of myocardium reflect features of cardiac pathogenesis in patients with COVID-19.

In addition to myofibril disruption, we also identified a lack of nuclear chromatin staining in many CMs after SARS-CoV-2 exposure, which was also observed in autopsy specimens from patients with COVID-19. Whether this observation reflects a generalized cytotoxic response, or specific SARS-CoV-2 induced disruption to the structural integrity of the cell is unknown (*50*). However, transcriptomic analysis suggests that SARS-CoV-2 disrupts expression of members of the nuclear LINC complex, through which the cytoskeleton supports the shape and structure of the nucleus (*51*, *52*). It is possible that SARS-CoV-2 could weaken the nuclear envelope, a process that could be exacerbated by post-fixation processing resulting in loss of nuclear material. Understanding the mechanism by which aberrant nuclear phenotypes arise will be crucial to determine if they are a clinical feature of COVID-19 in the heart.

Aside from myocytes in the heart, non-myocyte cardiac cells also mediate some of the observed outcomes in COVID-19, such as cardiac hypertrophy and vascular dysfunction (*33*), and in vivo infection of ECs has been reported (*53*, *54*). In our studies, CFs and ECs were not expected to be infectable due to their low ACE2 expression. qPCR of CFs and ECs exposed to SARS-CoV-2 supported these predictions and displayed no replicating virus, although potent cytopathic effects in both ECs and CFs were observed. Heat inactivated virus failed to recapitulate this cytotoxic effect, suggesting that abortive infection or paracrine signaling from a small population of infected cells may be responsible for inducing cell death. Future studies identifying specific mechanisms of viral toxicity to the cardiac stroma will be useful to determine how these cells may contribute to SARS-CoV-2-induced cardiac dysfunction.

The ability of iPSC-CMs to model the cardiopathic consequences of infectious pathogens opens a wide array of potential avenues for discovery and validation of candidate cardioprotective therapies for COVID-19 and other diseases. For example, our finding that SARS-CoV-2 infects CMs via an endolysosomal route may indicate that clinical trials which target TMPRSS2 to prevent COVID-19 (*55*, *56*) may not afford effective cardioprotection without orthogonal targeting of endosomal proteases (*57*). Additionally, although cell-based drug screens exist for many pathogens, including SARS-CoV-2 (*58*), the unique cytoarchitecture of cardiomyocytes and the specific effects of SARS-CoV-2 on these cells offer distinct screening possibilities. Identification of efficacious cardioprotective therapies may require preventing viral replication and maintaining sarcomeric integrity to achieve optimal therapeutic benefit.

Our study is limited by the inability to observe the progression of viral infection of cardiomyocytes, both in vitro (due to technical limitations of the Biosafety Level 3 used for this work) and in tissues of patients with COVID-19 (due to sample availability). Although our results show that direct infection of CMs may not be required to elicit cytotoxic effects in cardiac tissue, there is an increasing body of evidence supporting cardiomyocytes being directly infected by SARS-CoV-2 in patients with COVID-19 (*21*, *22*, *59*). SARS-CoV-2 can spread from lung epithelia to other organs through plasma, and viremia in COVID-19 has been closely associated with cardiac damage (*60*). Observing viral particles in autopsy tissue has been well documented in SARS-CoV and SARS-CoV-2 infection (*61*, *62*). However, if virus is only present in myocytes during an early progressive stage of the disease, inspection of deceased patient tissue may be well beyond the window of opportunity to detect transient viral presence in CMs. This hypothesis is supported by the observation that SARS-CoV-2 within myocytes has only been observed in patients who suffer rapid demise during acute viral illness (*21*, *59*, *63*). Similarly, our in vitro model of CMs infection allows for controlled study of the progression of the myocardial damage caused by SARS-CoV-2, thereby recapitulating earlier stages of infection. This model for infection is not free of limitations: iPSC-derived CMs are generally considered immature and may not fully recapitulate features of the adult heart of patients. In addition, our cultures may not mimic the circulating cytokine milieu resultant from infection of other organs (*20*, *64*), such as the lungs, in addition to lacking monocytes, which infiltrate SARS-CoV-2 infected tissues in patients. However, our CMs do express the main receptor ACE2 more than undifferentiated tissue or other cell types, in agreement with other adult heart studies (*16*, *18*). In addition, although our experiments model direct infection, we have also observed infection-independent cytopathic effects that may be able to recapitulate indirect effects of infection to non-infected cells. The phenotype observed in infected iPSC-CMs is reminiscent of our autopsy samples, making this a valuable system to study SARS-CoV-2 infection in the heart.

Reports of cardiac dysfunction incidence in individuals with COVID-19 range from 20% to 50%, independent of disease severity (*8*, *13*, *49*). Cardiac damage is strongly associated with disease mortality (*3*, *8*, *20*, *22*, *65*). In addition, due to the heart’s innate lack of regenerative capacity (*66*, *67*), a large fraction of patients with cardiac damage could suffer long-term cardiac sequelae from COVID-19. Our studies, which are analogous to mild cases of COVID-19 due to the low viral load that spreads through CMs in a short time frame, display signs of striking cytopathic effects similar to those we observe in patient samples without any clinical diagnosis of cardiac damage. These findings provide insight into the mechanism of cardiac pathology of COVID-19, and we anticipate they can help guide the development of efficacious anti-viral and cardioprotective therapies to help manage and prevent heart damage in patients with COVID-19.

## MATERIALS AND METHODS

### Study design

The goal of our study was to evaluate the SARS-CoV-2 infectivity of different heart cell types using of iPSC-derived tissue, and to compare the resulting cytopathic effects to heart autopsy samples from COVID-19 patients. Our starting hypothesis was that SARS-CoV-2 would be able to infect iPSC-CMs (and potentially iPSC-EC or iPSC-FC), and that viral infection could result in cytopathic features and toxicity. For in vitro infection experiments, iPSC-derived heart cells (mostly cardiomyocytes) were seeded in multi-well plates, pretreated with different drugs (where required), and taken into the Biosafety Level 3 (BSL-3) for infection with SARS-CoV-2 or other human CoV, or no infection at all (mock). Well contents were blinded before infection and until data (qPCR or imaging) quantification was completed. All conditions were performed with a minimum of triplicate measurements. In addition, qPCR measurements were done in technical triplicate. For staining, all images were obtained from a total of 10 randomly acquired fields of view per well. Nuclei counting was performed automatically using an unbiased algorithm. Sarcomere fragmentation and infectivity were manually annotated by three separate, blinded human operators, with a 10% image overlap across them to evaluate consistency. For the RNA-seq transcriptome analysis, samples were prepared in biological triplicates and validated by unbiased clustering of individual data points. All investigated gene expression changes and pathways enriched were statistically significant (*P <* 0.05*)* using appropriate statistical tests that account for multiple testing. For patient autopsy sample analysis, specimens from COVID-19 patients and non-COVID-19 individuals were blinded and processed for visual inspection. For each patient, images were acquired from 2-3 heart regions (right and left ventricles and interventricular septum) per sample, with 5-15 images per section/region. The research protocol for evaluation of autopsy specimens was approved by institutional review boards at the University of California, San Francisco (UCSF)/Zuckerberg San Francisco General Hospital (ZSFG) (IRB no. 20-31641). More details on the methodology can be found in this section and in the Supplementary Materials and Methods. A full list of materials and reagents used in this study can be found in **table S1.**

### hiPSC maintenance; iPSC-cardiomyocyte differentiation and purification

Human iPSCs [WTC line (*68*)] were maintained in mTESR or mTESR+ (STEMCELL Technologies) on Matrigel (8 μg/ml, BD Biosciences)-coated cell culture plates at 37°C, 5% CO_2_. Cells were passaged every 3 days using Relesr (STEMCELL Technologies) and supplemented with Rock Inhibitor Y-27632 (SelleckChem) for 24 hours after each passaging. hiPSCs were differentiated into cardiomyocytes following a modified Wnt pathway modulation-based GiWi protocol (*69*). Briefly, hiPSC cultures were harvested using Accutase (STEMCELL Technologies) and seeded onto Matrigel-coated 12-well plates. Three days later, cells were exposed to 12 uM CHIR99021 (Tocris) in R/B- [RPMI1640 (Gibco, 11875093) supplemented with B27 without insulin (Gibco, A1895601)] for 24 hours. After an additional 48 hours, media was changed to R/B- supplemented with 5 uM IWP2 (Tocris) for 48 hours. On day 7, media was changed to R/B+ [RPMI1640 medium supplemented with B27 with insulin (Gibco, 17504044)] and refreshed every 3 days thereafter. Beating was generally observed around day 8-11. At day 15, cells were cryopreserved using CryoStor CS10 (STEMCELL Technologies). After thawing, cell cultures were enriched for iPS-cardiomyocytes following metabolic switch purification (*70*). Briefly, cells were washed once with saline buffer and incubated in DMEM (without glucose, without sodium pyruvate; Gibco, 11966025) supplemented with GlutaMax (Gibco, 35050061), MEM Non-Essential Amino Acids (Gibco, 11140050) and sodium L-lactate (4 mM, Sigma-Aldrich). Lactate media was refreshed every other day for a total of 6 days. Four to six days later (day 28-30), iPS-CMs were replated into assay plates for infection using 0.25% Trypsin (Gibco, 15050065) at a density of about 60,000 cells/cm^2^.

### scRNAseq analysis of SARS-CoV-2 entry factors

A historic single cell RNA sequencing data set consisting of iPSC-derived cardiomyocytes, primary fetal cardiac fibroblasts, and iPSC-derived endothelial cells was re-analyzed to compare relative expression of SARS-CoV-2 relevant receptors and proteases (GSE155226) (*71*). Briefly, day 30 lactate-purified cardiomyocytes were force aggregated either alone or with a single supporting cell type and cultured in suspension culture. Aggregates were dissociated and libraries prepared using the Chromium 3′ v2 library preparation platform (10X Genomics). Libraries were sequenced on a NextSeq 550 sequencer (Illumina) to a depth of at least 30 million reads per sample. Samples were demultiplexed and aligned to GRCh38 with CellRanger v3.0.2. Individual cell unique molecular identifiers (UMIs) were filtered using Seurat v3.2.0 (*72*), keeping only cells with at least 1,000 reads, 300 detected genes, and less than 10% mitochondrial reads. The top 2,000 variable genes were projected onto 20 principal components. Although greater than 5% of cells were detected in either S or G2M phase, regressing out cell cycle genes did not alter clustering of primary cell types. Cells were clustered with a resolution of 0.4, yielding three primary clusters corresponding to each cell type, which were used to profile cell-type specific expression of SARS-CoV-2 relevant factors.

### SARS-CoV-2 and other human coronaviridae infections

The WA-1 strain (BEI resources) of SARS-CoV-2 was used for all experiments. All live virus experiments were performed in a Biosafety Level 3 lab. SARS-CoV-2 stocks were passaged in Vero cells (ATCC) and titer was determined via plaque assay on Vero cells as previously described (*73*). Briefly, virus was diluted 1:10^2^-1:10^6^ and incubated for 1 hour on Vero cells before an overlay of Avicel and complete DMEM (Sigma Aldrich, SLM-241) was added. After incubation at 37°C for 72 hours, the overlay was removed and cells were fixed with 10% formalin, stained with crystal violet, and counted for plaque formation. SARS-CoV-2 infections of iPSc and iPS-derived cardiac cells were done at a multiplicity of infection of 0.006 for 48 hours unless otherwise specified. For heat inactivation, SARS-CoV-2 stocks were incubated at 85°C for 5 min. For cell type specific viral titers, input virus was washed at 24 hours post infection, supernatants were collected at 48 hours and stored at -80 until plaque assays were performed.

All viral stocks were fully sequenced to monitor for acquisition of mutations, particularly in the furin active cleavage site. Viral stocks were prepared for sequencing using the Primal-Seq Nextera XT version 2.0, using the ARTIC Network V3 primers and sequenced on the Ilumina NovaSeq platform to an average read depth of >1000X coverage. Single nucleotide variants (SNVs) were called if they appeared at a >70% frequency. SARS-CoV-2/human/USA/USA-WA1/2020, complete genome (NCBI accession id MN985325.1) was used as the reference sequence. Three SNVs were detected that met this criteria: C5457T, T21874G, C23525T.

For infection with human coronaviruses NL63 and OC43, HCoV-NL63 virus culture Isolate Amsterdam I of HCoV-NL63 (NR-470, BEI Resources) was propagated in Huh7.5.1-ACE2 cells. Supernatant was harvested 5 days post infection, filtered and stored at -80°C. HCoV-OC43 (VR-1558, ATCC) was propagated in Vero E6 cells. Supernatant was harvested 6 days post infection, filtered and stored at -80°C. Cardiomyocyte infections were performed in a Biosafety Level 2 lab, at an MOI of 0.01. Cells were harvested for qPCR using Qiagen RLT buffer or fixed using 4% paraformaldehyde for subsequent analyses.

### Immunocytochemistry

Infected and mock-treated cell cultures in coverslips were washed with Phosphate Buffered Solution (PBS, Corning) and fixed in 4% paraformaldehyde (PFA) overnight, followed by blocking and permeabilization with 0.1% Triton-X 100 (T8787, Sigma) and 5% BSA (A4503, Sigma) for one hour at RT. Antibody dilution buffer (Ab buffer) was comprised of PBS supplemented with 0.1% Triton-X 100 and 1% BSA. Samples were incubated with primary antibodies overnight at 4°C **(table S2)**, followed by 3 washes with PBS and incubation with fluorescent-conjugated secondary antibodies at 1:250 in Ab buffer for 1 hour at RT **(table S2)**. Coverslips were mounted onto SuperFrost Slides (FisherBrand, 12-550-15) with ProLong Antifade mounting solution with DAPI (Invitrogen, P36931). Images were acquired with a Zeiss Axio Observer Z.1 Spinning Disk Confocal (Carl Zeiss) or with an ImageXpress Micro Confocal High-Content Imaging System (Molecular Devices) and processed using ZenBlue and ImageJ.

### RT-qPCR

Cultured cells were lysed with Qiagen buffer RLT (Qiagen, 79216) supplemented with 1% β-mercaptoethanol (Bio-Rad, 1610710) and RNA was isolated using the RNeasy Mini Kit (Qiagen 74104) or Quick-RNA MicroPrep (ThermoFisher, 50444593) and quantified using the NanoDrop 2000c (ThermoFisher). Viral load was measured by detection of the viral Nucleocapsid (N) transcript through one-step quantitative real-time PCR, performed using PrimeTime Gene Expression Master Mix (Integrated DNA Technologies, 1055772) with primers and probes specific to N (‘N5′) and RPP30 as in internal reference. RT-qPCR reactions were performed on a CFX384 (BioRad) and delta cycle threshold (ΔCt) was determined relative to RPP30 transcript amounts. Viral genomic RNA detection in pharmacologically treated samples were normalized to DMSO-treated controls. A full list of primers and probes used in this study can be found in **table S3.**

### RNA-Seq

To generate libraries for RNA-sequencing, RNA isolate quality was assessed with an Agilent Bioanalyzer 2100 on using the RNA Pico Kit (Agilent, 5067-1513). 10 ng of each RNA isolate was then prepared using the Takara SMARTer Stranded Total RNA-Seq Kit v2 – Pico Input Mammalian (Takara, 634412). Transcripts were fragmented for 3.5 min and amplified for 12 cycles. Library concentrations were quantified with the Qubit dsDNA HS Assay Kit (Thermo Fisher, Q32851) and pooled for sequencing. Sequencing was performed on an Illumina NextSeq 550 system, using the NextSeq 500/550 High Output Kit v2.5 (150 Cycles) (Illumina, 20024907) to a depth of at least 10 million reads per sample. Raw data are available at GEO under the accession number GSE156754.

### Statistical analyses

Statistical testing for qPCR experiments was performed using GraphPad Prism 8 software, using 1-way ANOVA with post-hoc Tukey’s multiple comparisons test. Statistical analysis for the immunofluorescence cell counts was performed using R*(74)* using Student’s *t* test with Bonferroni correction for multiple testing. Statistical differences in expression between bioinformatic samples were performed on corrected, log transformed counts using Welch’s *t* test with Benjamini Hochberg false discovery rate correction with a threshold of 0.05, except where described above. Individual-level data are reported in data file S1.

## Supplementary Materials

stm.sciencemag.org/cgi/content/full/scitranslmed.abf7872/DC1 Materials and Methods Fig. S1. Expression of SARS-CoV-2 entry factors in iPSC-derived cardiac cells, productive infection assay and drug pretreatment effects. Fig. S2. Transcriptional disruption and cellular pathway dysregulation due to SARS-CoV-2 exposure in cardiac cells. Fig. S3. Heat maps for gene pathways of interest, comparing transcriptional differences in mock and SARS-CoV-2-infected cardiomyocytes. Fig. S4. Cardiomyocyte infection by SARS-CoV-2 leads to dramatic transcriptomic disruption in genes related with the nuclear envelope and sarcomere structures. Fig. S5. iPSC-CM cultures infected with SARS-CoV-2 display myofibrillar fragmentation. Fig. S6. Myofibrillar fragmentation phenotype is partially recapitulated by proteasomal inhibition, but not by cardiotoxic drug doxorubicin or infection by coronavirus NL-63 or OC-43. Fig. S7. Additional representative images from heart autopsy samples from patients with and without COVID-19. Table S1. List of reagents. Table S2. List of dyes, primary and secondary antibodies used for immunocytochemistry and paraffin sections. Table S3. List of RT-qPCR primer and probe sequences. Data file S1. Individual-level data. References (*75*–*84*)
